# Kruppel-like factor 13 acts as a tumor suppressor in thyroid carcinoma by downregulating IFIT1

**DOI:** 10.1186/s13062-023-00422-5

**Published:** 2023-10-10

**Authors:** Yang Liu, Yixuan Song, Yuqin He, Ziren Kong, Han Li, Yiming Zhu, Shaoyan Liu

**Affiliations:** Department of Head and Neck Surgical Oncology, National Cancer Center/National Clinical Research Center for Cancer/Cancer Hospital, Chinese Academy of Medical Sciences and Peking Union Medical College, No. 17, Panjiayuan Nanli, Chaoyang District, Beijing, 100021 People’s Republic of China

**Keywords:** Thyroid carcinoma, KLF13, IFIT1, Cell proliferation

## Abstract

**Background:**

Kruppel-like factor 13 (KLF13) is a transcription factor and plays an important role in carcinogenesis. However, the significance of KLF13 in thyroid carcinoma (THCA) is underdetermined. In this study, we aimed to explore the clinical relevance and function of KLF13 in the progress of THCA.

**Methods:**

The expression of KLF13 in thyroid carcinoma and normal tissue was investigated by qPCR and IHC assay. The expression of KLF13 and IFIT1 in cell samples was investigated with Western blot assay. Cell proliferation ability was detected with CCK8 and colony formation assay. Cell growth in vivo with or without KLF13 overexpression was evaluated on a xenograft model. Cell migration ability was measured with Transwell assay. Cell cycle was detected with flow cytometer. The downstream genes of KLF13 were screened using RNA-seq assay. Luciferase activity was employed to assess the transcriptional regulation of KLF13 on IFIT1 promoter.

**Results:**

KLF13 expression was downregulated in THCA samples. KLF13 knockdown and overexpression promoted and inhibited the proliferation and migration of THCA cells, respectively. The RNA-seq, RT-qPCR and immunoblotting data showed that KLF13 knockdown significantly potentiated IFIT1 expression at both mRNA and protein levels. Luciferase assays showed that KLF13 suppressed the transcription activity of IFIT1 promoter. Besides, IFIT1 upregulation was critical for the proliferation and migration of THCA cell lines. Lastly, silencing of IFIT1 greatly reversed the proliferation and migration induced by KLF13 knockdown.

**Conclusions:**

In conclusion, KLF13 may function as an anti-tumor protein in THCA by regulating the expression of IFIT1 and offer a theoretical foundation for treating thyroid carcinoma.

**Supplementary Information:**

The online version contains supplementary material available at 10.1186/s13062-023-00422-5.

## Background

Thyroid cancer (THCA) is in charge of 586,000 cases across the world, ranking the 9th place for incidence in 2020 [1]. Most thyroid cancers can be divided into two categories according to their source cells: the vast majority (above 95%) come from follicular cells, while the surplus 3–5% are medullary thyroid cancers brought by C cells [2]. Follicular-cell-derived carcinomas can be fallen into anaplastic thyroid carcinoma (ATC), Hurthle cell carcinoma, papillary thyroid carcinoma, poorly differentiated thyroid carcinoma, and follicular thyroid carcinoma [3]. The most common treatment options include surgery, radioactive iodine therapy, radiotherapy, systemic therapy, and personalized medicine, and are chosen according to the patient’s condition [4, 5].

Kruppel-like factors (KLFs), a large family of transcription factors, have important roles in cell growth, cell differentiation, and early embryonic growth [6, 7]. According to their transcriptional activity, KLF 1 to 17 can be classified into KLF1, 2, 4, 5, 6, and 7, which have “activator” effects; and the remaining ones, which have inhibitory effects. The roles of KLFs have been widely researched in different cancers. KLF1, like KLF3 and KLF8, can function as a proto-oncogene in many cancers. On the contrary, KLF2, KLF4, and KLF6 are anti-tumor factors in most cancers [8].

Kruppel-like factor 13 (KLF13) has three classical zinc-finger DNA-binding regions and consists of two cysteine and two histidine (C2H2 motif) tetrahedral coordinated zinc atoms. It binds to GC-rich sequences and associated GT and CACCC boxes [9].The function of KLF13 on cancer progression are diverse in different cancers. KLF13 functions as a tumor suppressor protein in prostate carcinoma [10], pancreatic cancer [11, 12], colorectal cancer [13], glioma [14, 15] and clear cell renal cell carcinoma [16], while it functions as a oncogene in hepatocellular carcinoma [17, 18] and oral cancer [19]. However, the previse role of KLF13 in THCA was unclear and needs experiments to illustrate.

In this study, we aim to explore the clinical significance, function and underlying mechanisms of KLF13 in THCA progression. We showed that KLF13 acted as an anti-tumor factor since it was downregulated in THCA samples and retarded the proliferation and migration of THCA cells. Mechanistically, KLF13 repressed the transcription activity and expression of IFIT1, which was critical for the accelerated growth and migration of TCHA cells triggered by downregulation of KLF13. An inverse correlation between KLF13 and IFIT1 was found in THCA patients. Our study demonstrates that KLF13 suppresses the progression of THCA via negative regulation of IFIT1.

## Results

### Downregulation of KLF13 in thyroid carcinoma

**Fig. 1:**
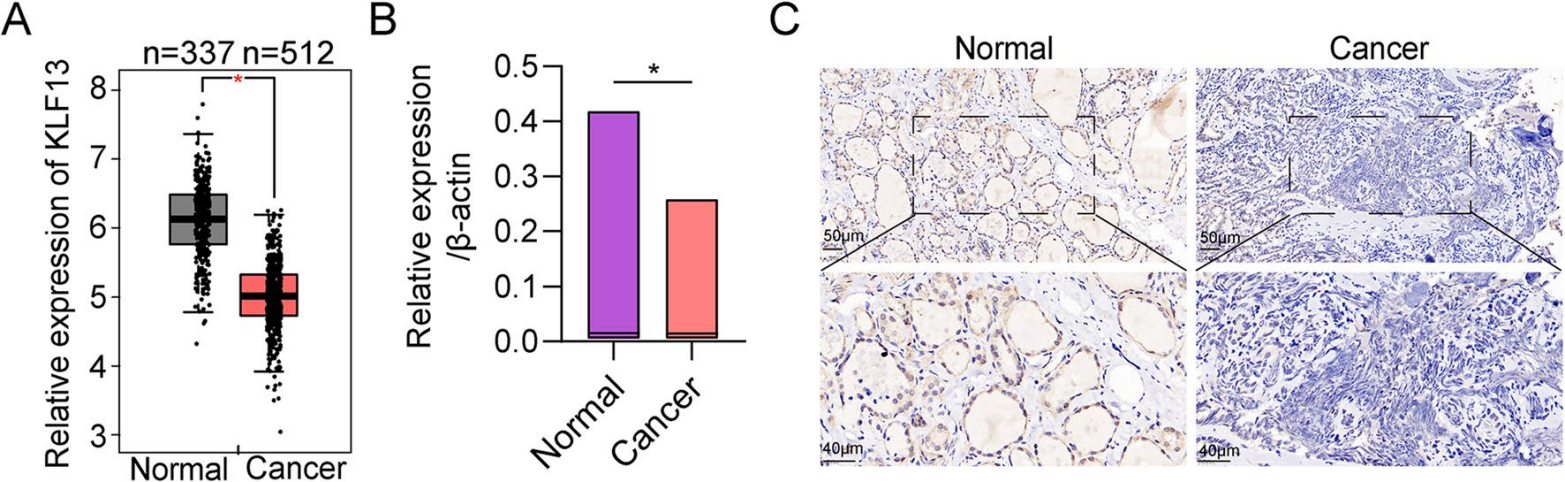
Downregulation of KLF13 in thyroid carcinoma. **A** KLF13 expression in normal samples (*n* = 337) and thyroid carcinoma samples (*n* = 512) from TCGA. **B** KLF13 expression in normal tissue and thyroid carcinoma by qRT-PCR. **C** Typical IHC staining of KLF13 in thyroid carcinoma and their nearby non-tumor tissues (*n* = 25). *P < 0.05

To explore the role of KLF13 in thyroid carcinoma, the expression of KLF13 was analyzed in TCGA. Compared with normal samples, KLF13 was downregulated in thyroid carcinoma (Fig. 1A). This finding was further confirmed by qRT-PCR, which showed that KLF13 mRNA levels in thyroid carcinoma were lower than those in normal samples (Fig. 1B). To verify these results, IHC staining of KLF13 in 25 pairs of human thyroid tissues was made. IHC analysis showed a declined expression of KLF13 in THCA tissues (Fig. 1C and Table 1). These results indicate that KLF13 might be a tumor suppressing gene.

**Table 1:** The protein abundance of KLF13 in thyroid cancer and normal tissues by IHC

	Cancer	Normal	*χ*^2^	*P* Value
High expression	5	17	11.69	< 0.001
Low expression	20	8		
Total	25	25		

### Downregulation of KLF13 promotes cell propagation, migration, and cell cycle

**Fig. 2:**
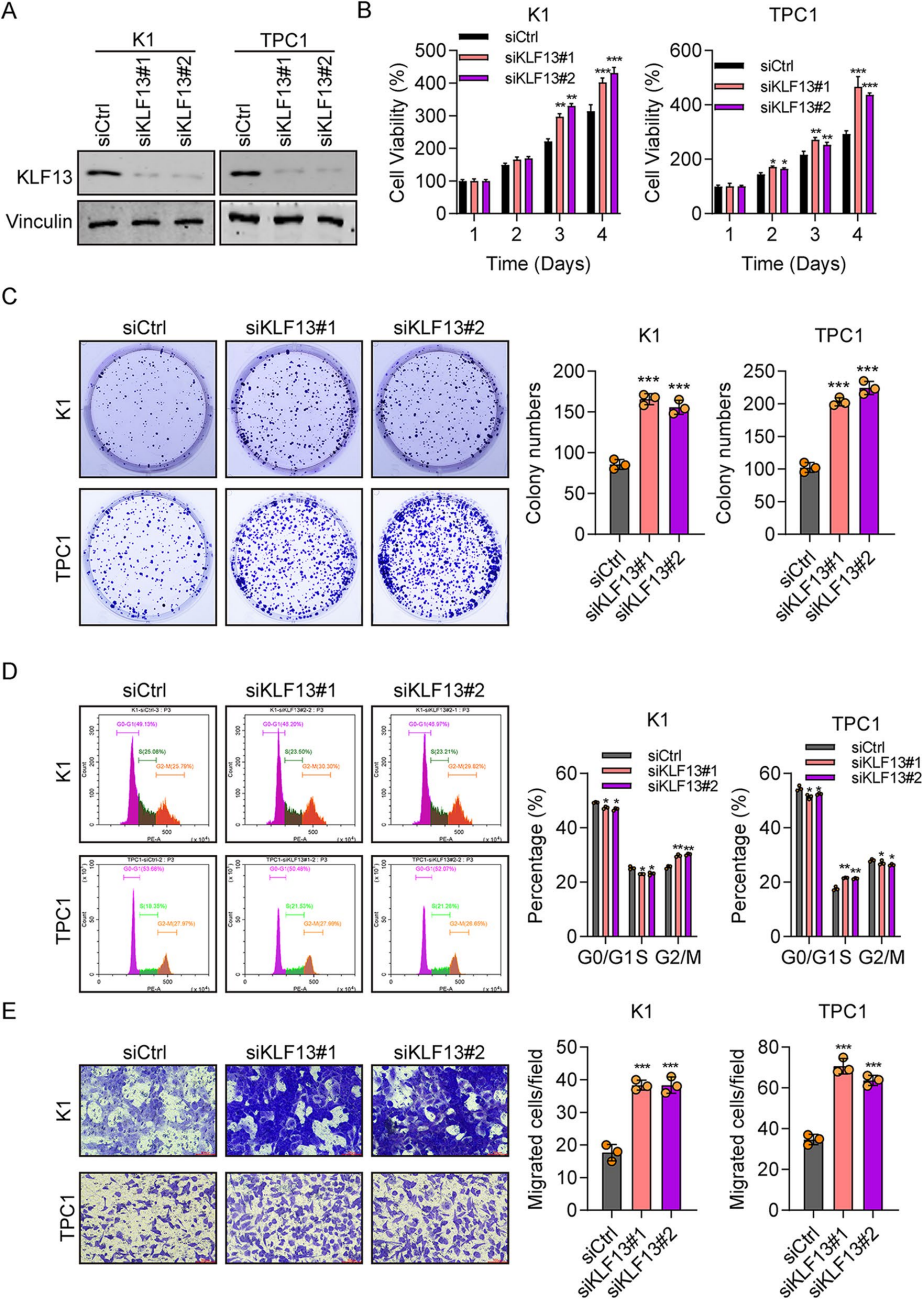
Downregulation of KLF13 promotes cell propagation, migration, and cell cycle. **A** Efficiency of KLF13 knockdown in K1 and TPC1 cells through western blot. **B** Propagation of K1 and TPC1 cells transfected with siCtrl, siKLF13#1, or siKLF13#2 by CCK8 analysis. **C** Colonies formed in cells transfected with siCtrl, siKLF13#1, or siKLF13#2. **D** Cell cycle analysis in siCtrl-, siKLF13#1-, and siKLF13#2-transfected cells by flow cytometry. **E** Percentage of migrating K1 and TPC1 cells transfected with siCtrl, siKLF13#1, or siKLF13#2 by transwell migration assay. Vinculin was used as a loading control. *P < 0.05, **P < 0.01, ***P < 0.001

Next, we studied the function of KLF13 by using siRNAs. The western blot showed that KLF13 expression was markedly declined in siKLF13-transfected cells (Fig. 2A). CCK8 results showed that KLF13 depletion promoted the proliferation in both cell types (Fig. 2B). More colonies were formed in siKLF13-transfected cells by comparing with the siCtrl group (Fig. 2C). Flow cytometry results displayed that knockdown of KLF13 decreased the proportion of G0/G1 phase cells (Fig. 2D). By comparing with the control group, cell migration with KLF13 knockdown was greatly increased (nearly doubled in both two cell types) (Fig. 2E). These results displayed that the progression of the cell cycle was adversely affected by KLF13 downregulation, which further suggests the tumor suppressor role for KLF13.

### KLF13 overexpression suppresses cell proliferation, migration, and cell Cycle

**Fig. 3:**
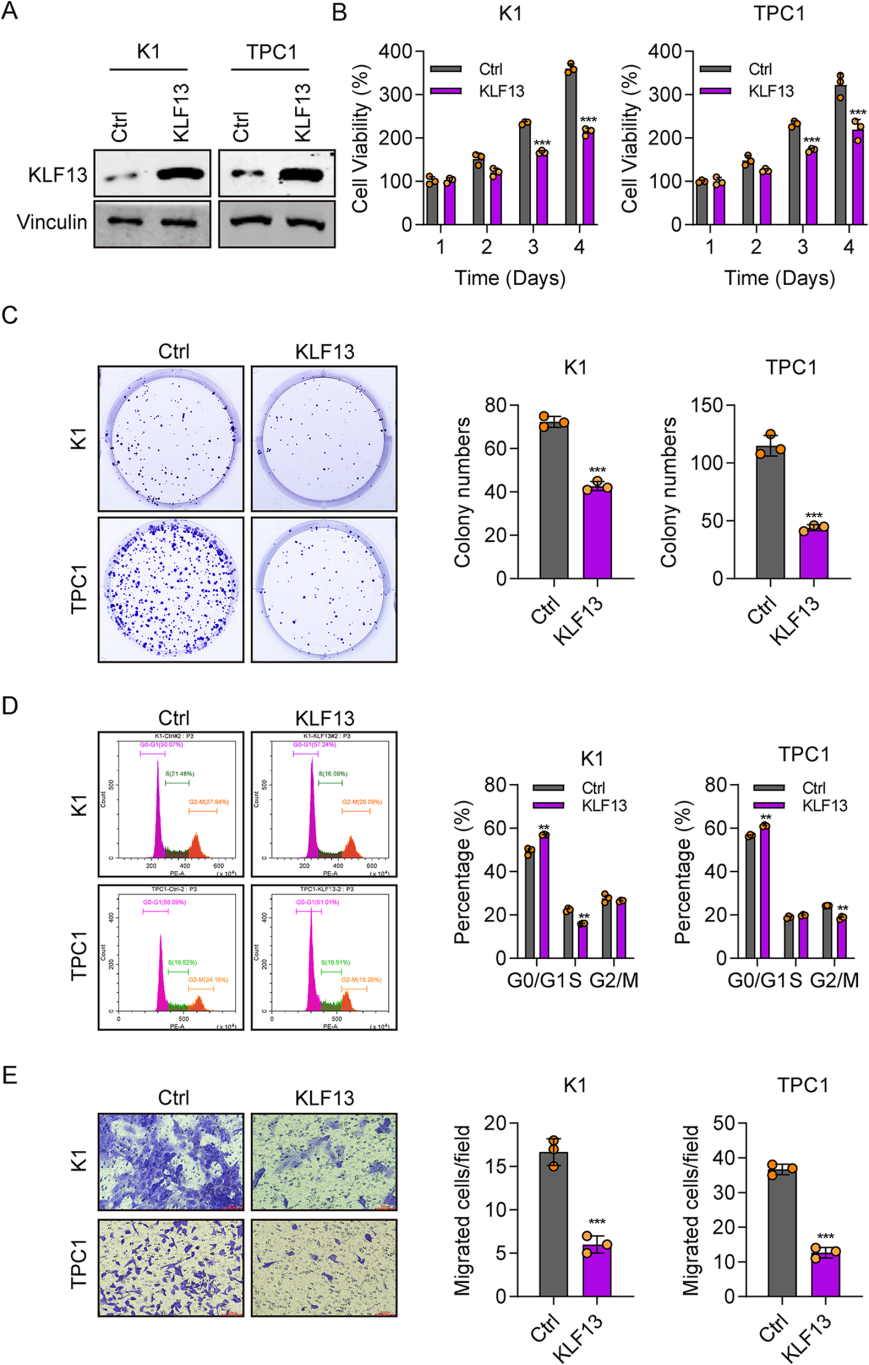
KLF13 overexpression suppresses cell proliferation, migration, and cell cycle. **A** KLF13 overexpression in K1 and TPC1 cells through western blotting. **B** Cell viability in control or KLF13-overexpressing cells as measured by CCK8. **C** Colony formation assay outcomes for control and KLF13-overexpressing cells. **D** Cell cycle measured by flow cytometry for control and KLF13-overexpressing K1 and TPC1 cells. **E** Transwell migration analysis of control and KLF13-overexpressing cells. Vinculin was used as a loading control. **P < 0.01, ***P < 0.001

To validate the findings, we performed KLF13 overexpression in THCA cells. Western blot displayed that KLF13 levels were greatly higher than those in the control group (Fig. 3A). From day 1 to 4, cell viability of KLF13-overexpressing cells gradually decreased (Fig. 3B). Colony formation ability (Fig. 3C) and transwell migration numbers (Fig. 3E) were also decreased in the KLF13-overexpression group. Flow cytometry outcomes showed that KLF13 overexpression suppressed the progression of the cell cycle, with increased percentage of G0/G1-stage cells (Fig. 3D). These outcomes further determined the tumor suppressor effect of KLF13 on THCA.

### KLF13 suppresses tumor growth of THCA in vivo

**Fig. 4:**
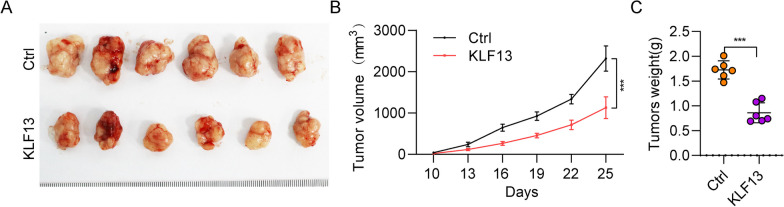
KLF13 suppresses tumor development in vivo of thyroid cancer. **A** The figures of tumor size in nude mice between two groups. **B** Tumor volume between two groups. **C** Tumor weight between two groups. P*** < 0.001

As demonstrated above that KLF13 suppresses cell propagation and migration of THCA cells, we next examined this role of KLF13 in tumor development in vivo using tumor xenograft models. The results showed that overexpression of KLF13 greatly decreased the tumor size (Fig. 4A), volume (Fig. 4B) and weight (Fig. 4C), indicating that KLF13 suppressed the tumor growth of THCA.

### KLF13 Negatively Regulates the Expression of IFIT1

**Fig. 5:**
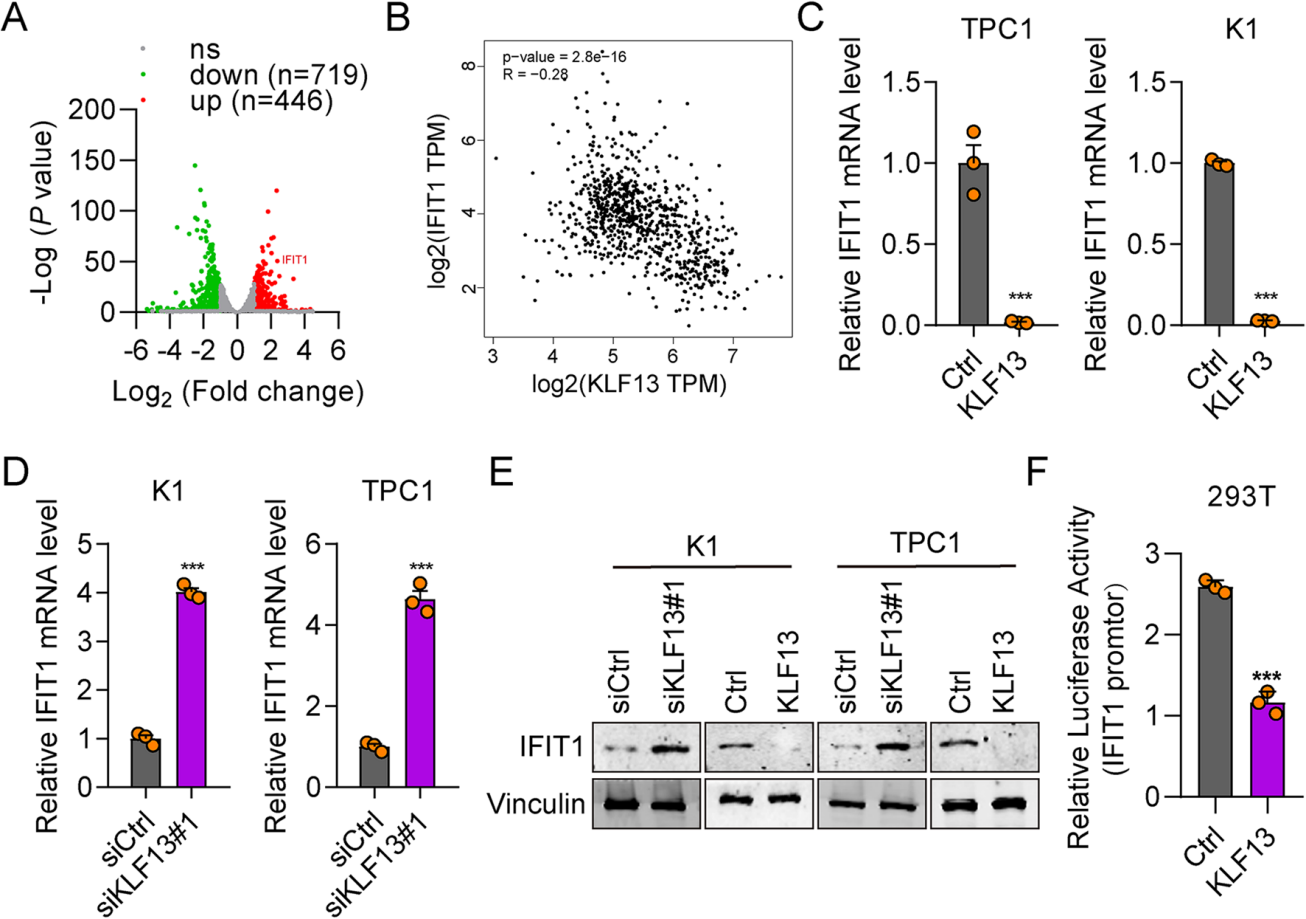
KLF13 negatively regulates IFIT1 expression in THCA cells. **A** Differentially expressed genes in siKLF13 cells as compared with siCtrl cells. Genes upregulated and downregulated were displayed as red and green dots. **B** Correlation between KLF13 and IFIT1 expression in human THCA samples based on TCGA database (*n* = 512). *R* = 0.28. *P* < 0.0001. **C**, **D** RT-qPCR results of IFIT1 after KLF13 knockdown or overexpression in K1 and TPC1 cells. ***P < 0.001. **E** Immunoblotting results of IFIT1 after KLF13 knockdown or overexpression in K1 and TPC1 cells. **F** Luciferase activity was measured to analyze the regulation of KLF13 on the transcription activity of IFIT1 promoter. ***P < 0.001

To explore the underlying mechanisms of KLF13 in THCA, we performed RNA sequencing in K1 cells transfected with siCtrl and siKLF13. KLF13 knockdown resulted in 719 genes downregulation and 446 genes upregulation (Fig. 5A). Since KLF13 is a transcription repressor gene, we focused on the upregulated genes regulated by knockdown of KLF13. Then we analyzed the correlation between the KLF13 and top 20 upregulated genes after KLF13 knockdown in the TCGA database. The results showed that the IFIT1 was the most negatively correlated gene with KLF13 (R = -0.28, p = 2.8E-16), as compared with TXNDC5, ERVV-2, ESM1, MMP13 and CCL20 (Additional file 1: Figure S1 and Fig. 5B). To verify the correlation between KLF13 and IFIT1 in TCHA, we performed immunohistochemistry assays in THCA samples derived from the same patients. The results demonstrated that there was a negative correlation between KLF13 and IFIT1 in THCA samples (Additional file 1: Figure S2 and Additional file 2: Table S1). Previous studies have reported that IFIT1 acts as an oncogene in various tumors [20–22], whereas its significance, as well as its involvement in KLF13 suppression of tumor progression, in THCA remains to be determined. Thus, we further studied the regulation of KLF13 on the transcription activity of IFIT1. The relative mRNA level of IFIT1 was significantly increased in siKLF13#1 transfected K1 and TPC1 cells, while greatly decreased in KLF13 overexpressed thyroid cancer cells (Fig. 5C-D). Similar results were observed on the protein expression of IFIT1 as shown by the western blot results (Figs. 5E). Luciferase activity assays further confirmed that KLF13 negatively modulated the transcription activity of the promoter of IFIT1 in 293 T cells (Fig. 5F). These results suggest that KLF13 negatively regulates the expression of IFIT1 in THCA cells and patients.

### IFIT1 promotes cell propagation and migration of Thyroid Cancer

**Fig. 6:**
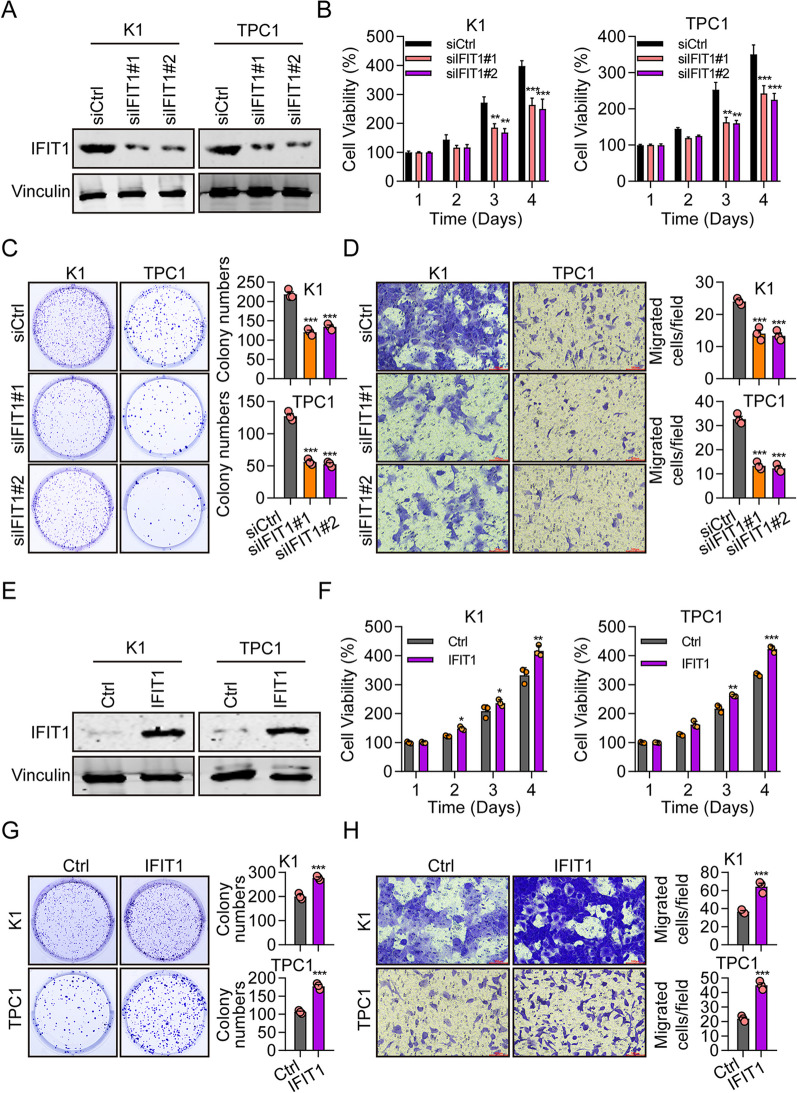
IFIT1 promotes the growth and migration of THCA cells. **A** The protein levels of IFIT1 in K1 and TPC1 cells transfected with siCtrl, siIFIT1#1, or siIFIT1#2 were measured by western blotting. **B** Cell viability of K1 and TPC1 cells transfected with siCtrl, siIFIT1#1, or siIFIT1#2 was detected by CCK8 assay. **C**, **D** Cell colony formation **C** and migration abilities (**D**) of K1 and TPC1 cells transfected with siCtrl, siIFIT1#1, or siIFIT1#2 were analyzed by transwell assay. **E** The protein levels of IFIT1 in K1 and TPC1 cells transfected with Ctrl, IFIT1 were detected by western blotting. **F** Cell viability of K1 and TPC1 cells transfected with Ctrl, IFIT1 was analyzed by CCK8 assay. **G**, **H** Cell colony formation (**G**) and migration abilities (**H**) of K1 and TPC1 cells transfected with Ctrl, IFIT1 were detected by transwell assay *P < 0.05, **P < 0.01, ***P < 0.001

To dig the effect of IFIT1 on thyroid carcinoma cells, we knockdown IFIT1 using siRNAs targeting to IFIT1. Western blot validated the knockdown effectiveness of IFIT1 in K1 and TPC1 cells (Fig. 6A). IFIT1 silencing significantly reduced cell viability (Fig. 6B) and cell colony formation numbers (Fig. 6C). Transwell assay results also showed the decreased migrated cells in IFIT1 knockdown cells (Fig. 6D). The overexpression efficiency of IFIT1 was also confirmed using western blot (Fig. 6E), and overexpression of IFIT1 greatly increased cell proliferation, colony, and migration in K1 and TPC1 cells (Fig. 6F–H). These findings indicate that IFIT1 may function as an oncogene in THCA.

### Silencing of IFIT1 reverses KLF13 knockdown-induced THCA cell propagation and migration

**Fig. 7:**
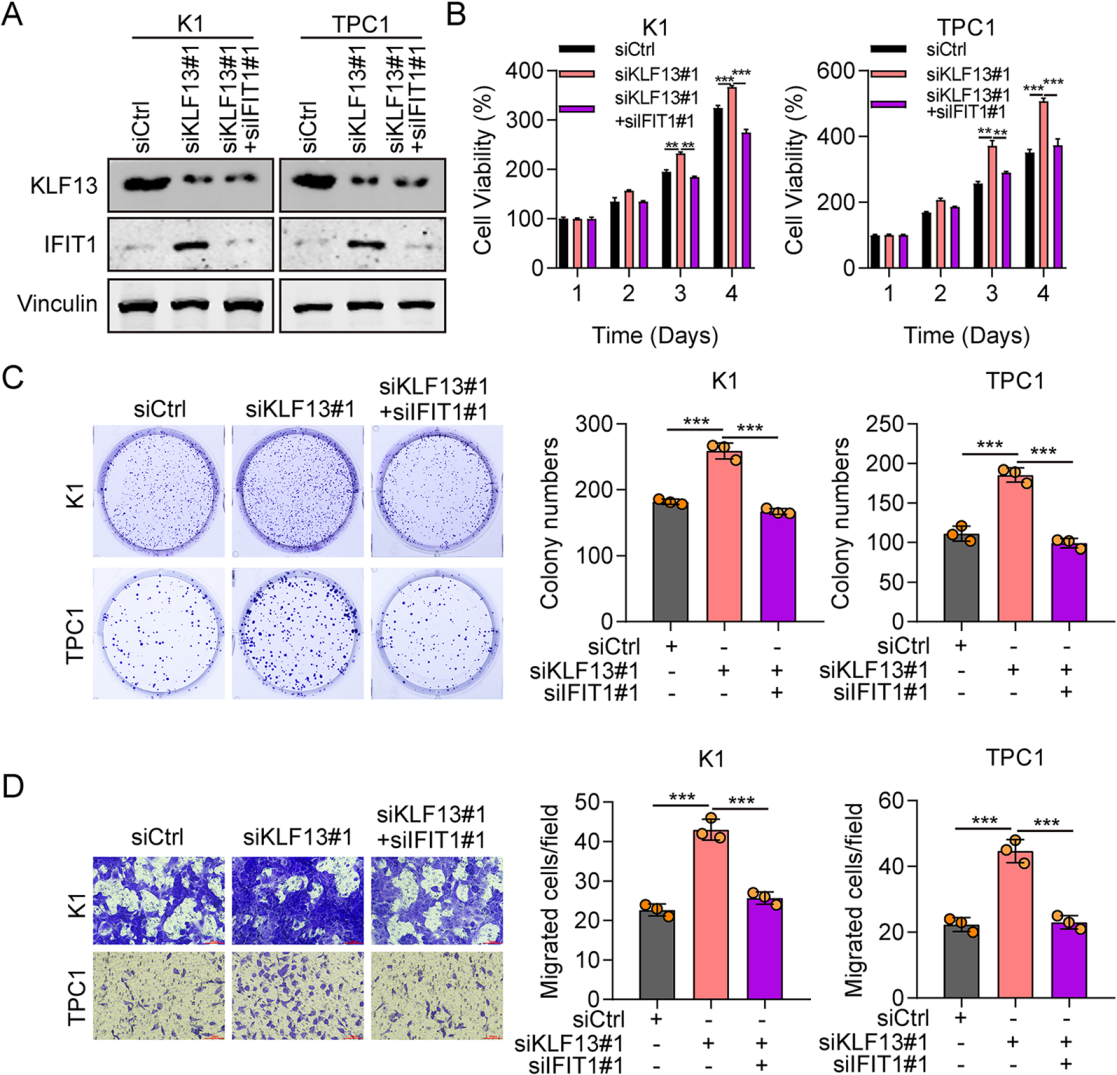
KLF13 downregulation promotes cell propagation and migration via upregulating IFIT1. **A** The expression of KLF13 and IFIT1 in K1 and TPC1 cells which are transfected with siCtrl, siKLF13#1, siKLF13#1 + siIFIT1#1 were detected using western blot. **B**, **C** CCK-8 and colony formation were performed to assess the role of siIFIT1#1 in THCA cell proliferation. **D** The roles of siIFIT1#1 in THCA cell migration were examined by transwell assay. **P < 0.01, ***P < 0.001

To investigate whether KLF13 knockdown promotes THCA cell function via IFIT1, we detected the cell viability, cell colony numbers and cell movement in K1 and TPC1 cells co-transfected with siKLF13#1 and siIFIT1#1. Western blot confirmed the knockdown effectiveness of IFIT1 in KLF13 knockdown K1 and TPC1 cells (Fig. 7A). In addition, CCK-8 and colony formation results demonstrated that siIFIT1#1 reduced cell viability that formerly promoted after KLF13 knockdown in K1 and TPC1 cell lines (Fig. 7B, C). Besides, siIFIT1#1 also significantly reduced cell migration in both THCA cell lines combined with downregulation of KLF13 compare to single KLF13 knockdown (Fig. 7D). Thus, KLF13 downregulation contributes to cell propagation and migration via IFIT1 in THCA.

## Discussion

The KLF family comprises 17 members with physiological and pathological roles. It was shown that KLF2 knockdown enhanced cell propagation and invasion and migration in thyroid carcinoma [23]. High expression of KLF4 in ATC cells is found, and the phenotype and drug resistance in vitro and in vivo shall be maintained [24]. KLF5 affects biological function and sensitivity to chemotherapy of ATC through the JNK signaling pathway [25]. *KLF9,* a target gene of TF4, can induce cell apoptosis in medullary thyroid carcinoma [26]. KLF12 knockdown was shown to enhance the inhibitory effect of miR-628-5p on PTC cell propagation and metastasis [27]. Moreover, inhibition of KLF17 expression could induce tumor cell proliferation and metastasis and was related to poor prognosis in PTC [28]. Nevertheless, the exact effect of KLF13 on thyroid carcinoma remained unclear. This paper showed that KLF13 expression was lower in THCA tissues by comparing with normal thyroid tissues. Downregulation or upregulation of KLF13 could promote or inhibit cell propagation, movement and cell cycle of K1 and TPC1 cells. Tumor xenograft model also confirmed the tumor suppressor role of KLF13 on tumor growth of THCA.

To further explore the mechanism of KLF13 on THCA development, we scanned the downstream gene of KLF13 using RNA-seq. There were 446 genes upregulated and 710 genes downregulated in KLF13 knockdown TPC1 cells compare to control cells. IFIT1 was one of the most significantly upregulated gene, which was verified to negatively correlate with KLF13 in THCA samples from TCGA database and IHC results (Additional file 1: Figure S1 and S2). IFIT1 is identified as an interferon-stimulated genes (ISGs) [29]. IFIT family members can mediate protein–protein interactions because they all contain multiple tetratricopeptide motifs, which can scaffold to form repeats [30]. IFIT1 is induced by type I interferon stimulation in the present of double‐stranded RNAs or viral infection [31]. High expression of IFIT1 was discovered in esophageal cancer cells, with possible correlations with prognosis [32]. Another study showed the overexpression of IFIT1 in head and neck squamous cell carcinoma, and there was correlation with bad prognosis and tumor-associated macrophage markers [33]. IFIT1 expression has been shown to be positively related to the expression of p-EGFR^Y1068^ in Oral Squamous Cell Carcinoma [22]. Therefore, we intended to explore whether KLF13 suppressed THCA progression via regulating the expression of IFIT1.

In our study, KLF13 expression was negatively correlated with IFIT1 expression. Luciferase assay demonstrated the interaction of KLF13 and the promoter of IFIT1 in THCA cells. Suppression of IFIT1 retarded the propagation and migration of K1 and TPC1 cells, while overexpression of IFIT1 exhibited the opposite results. The mRNA and protein level of IFIT1 was regulated by KLF13 expression in thyroid carcinoma. Recovery assay showed that IFIT1 knockdown alleviated the enhanced cell propagation and migration induced by KLF13 knockdown. These results indicate KLF13 regulated THCA development via IFIT1.

## Conclusions

We conclude that KLF13 exerts an anti-tumor effect by negatively regulating the expression of IFIT1 in THCA. Downregulation of KLF13 or upregulation of IFIT1 drove cell propagation and migration, accelerate cell cycle progression in K1 and TPC1 cells, while overexpression of KLF13 or knockdown of IFIT1 inhibited these processes. Overexpression of KLF13 also suppressed tumor growth in vivo. Thus, our work demonstrates that the tumor suppressor effect of KLF13 on thyroid carcinoma, which is mediated by regulation of IFIT1 expression through binding to its promoter region.

## Methods

### Clinical samples

A total of 25 thyroid cancer tissue and paired normal tissues were collected from the patients before receiving any therapy interventions from April 2020 to August 2020 at the Department of Head and Neck Surgical Oncology, Cancer Hospital, Chinese Academy of Medical Sciences and Peking Union Medical College. The research was approved by the Ethics Committee of National Cancer Center/National Clinical Research Center for Cancer/Cancer Hospital, Chinese Academy of Medical Sciences and Peking Union Medical College and conducted under the guidance of the Declaration of Helsinki. Informed consent regarding the use of specimens was obtained from all patients.

### Immunohistochemistry

Deparaffinized with xylene, the tumor tissue and normal tissues were rehydrated with alcohol gradient. Next, 30-min retrieval was carried out in 1 mM EDTA buffer at pH 8.0, and endogenous peroxidases was blocked. Then, anti-KLF13 and anti-IFIT1 antibodies were applied to incubate the tissues at 4℃ overnight, followed by 60-min incubation of HRP-conjugated secondary antibody at 25℃. After staining with diaminobenzidine substrate, the 3-min counterstaining of sections was made with hematoxylin, followed by mounting in neutral balsam, and investigation under a microscope.

### Cell culture and transfection

TPC1 and K1 cells were cultured in Dulbecco’s modified Eagle’s medium (DMEM, Gibco, Thermo Fisher Scientific), with 2 mM glutamine, 10% fetal bovine serum, and 1% penicillin/streptomycin. Cells were kept in a 37 °C cell incubator with 5% CO_2_.

siRNAs and overexpression plasmids were used for knockdown and overexpressing experiments. For knockdown assay, Hippo Biotechnology (Huzhou, Zhejiang, China) provided the small interfering RNAs (siRNAs) which were indicated below. siKLF13#1: 5′-GCGAGAAAGUUUACGGGAAAU-3′; siKLF13#2: 5′-CGGGCGAGAAGAAGUUCAGCU-3′; siIFIT1#1: 5′-CUUCGGAGAAAGGCAUUAGAU-3′; siIFIT1#2: 5′-CGUCAAUGCAAUUAUCCAUUA -3′; siCtrl: 5′-CAGUACUUUUGUGUAGUACAAA-3′. The siRNA transfection was conducted with Lipofectamine RNAiMAX transfection reagent. For overexpression assay, the coding sequence was cloned and inserted into the overexpression plasmids. Cells were transfected using Lipofectamine 3000 (Thermo Fisher Scientific).

### RNA Extraction and Quantitative Real-time PCR (qRT-PCR)

TRIzol (Thermo Fisher Scientific) was adopted to isolate total RNA. M-MLV-RTase (Promega) was used to reversely transcribe the RNA. The ReverTra Ace qPCR RT kit (TransGen Biotech) was used for qPCR on a real-time PCR system (Bio-Rad). The primers used for qRT-PCR were shown below. KLF13-F: 5′-CGGCCTCAGACAAAGGGTC-3′, KLF13-R, 5′-TTCCCGTAAACTTTCTCGCAG-3′; IFIT1-F: 5′-TTGATGACGATGAAATGCCTGA-3′, IFIT1-R, 5′-CAGGTCACCAGACTCCTCAC-3′; β-actin-F, 5′-CATGTACGTTGCTATCCAGGC-3′, β-actin-R, 5′-CTCCTTAATGTCACGCACGAT-3′.

### Western Blotting

RIPA buffer with proteinase inhibitors was employed for cell lysis. Bradford reagent (Sigma; Merck) was adopted to measure protein concentrations. A total 20-μg protein were separated on 10% sodium dodecyl sulfate polyacrylamide gel electrophoresis. Then the proteins were transferred onto nitrocellulose membranes, followed by 1-h blocking with 5% non-fat milk at room temperature. Antibodies against KLF13 (18352-1-AP, proteintech), IFIT1 (23247-1-AP, proteintech), and Vinculin (ab91459, abcam) were incubated with the membranes overnight at 4 °C, followed by incubation with secondary antibodies for 2-h at room temperature.

### CCK-8 assay

CCK-8 reagent was bought from Yeasen Biotechnology. Cell seeding (2000 per well) was made into 96-well plates for 24 h, 48 h, 72 h, or 96 h. Each group included four wells and four repeats. Then, CCK-8 reagent was put to cells with 2-h incubation at 37 °C. The measurement of the absorbance of every well was made at 450 nm.

### Colony formation assay

The seeding of 600 cells per well was made into six-well plates, with three repeats for every sample. The medium was replaced each 3 days. Colony generation was made after 14 days. Cell washing was conducted three times with phosphate-buffered saline, then 4% paraformaldehyde was adopted for colony fixture for 30 min, and 500 μL 0.1% crystal violet staining solution was employed for staining for 20 min.

### Cell cycle analysis

A Cell Cycle Kit (Thermo Fisher Scientific) was adopted to explore cell cycling. First, digested with trypsin, cells were centrifuged for 5 min at 13,000 rpm. Then, pre-cooled PBS buffer was applied for washing the cells, and 70% pre-cooled ethanol was employed for cell fixture for 1 h. Then, after washing in PBS, 30-min cell staining was made with 0.5 ml staining solution in the dark at room temperature. Cell analysis was made with a Guava easyCyte HT system (Millipore).

### Transwell assay

Cell seeding was performed in the upper chamber of a 24-well Corning ® FluoroBlok ™ Cell Culture Inserts. DMEM with the addition of 10% fetal bovine serum was adopted to fill the lower chamber as a chemical attractant. The measurement of cell migration was made. Cell staining was made with crystal violet (0.5%), and the counting of cells was made under an inverted fluorescence microscope.

### RNA sequencing

The extraction of total RNA from TPC1 cells was conducted with TRIzol reagent. Total RNA integrity was detected by agarose electrophoresis, and quality and quantity were measured with a NanoDrop 2000 spectrophotometer. A sequencing library was established with an RNA library construction kit. Illumina HiSeq™ 2000 system was used for sequencing and analysis.

### Bioinformatics analysis

The expression of KLF13 and IFIT1, as well as the correlation between KLF13 and IFIT1 in THCA patients were analyzed on The Cancer Genome Atlas (TCGA) database.

### Vector construction and luciferase assay

After amplification, the promoter sequence of the IFIT1 gene was cloned into a pGL4.10-report vector. pGL4.10-IFIT1-promoter and Renilla expression vector pRL-TK were co-transfected into 293 T cells. A Dual-Glo Luciferase Assay system was adopted for assessing the luciferase activity forty-eight hours post-transfection.

### Tumor Xenograft model

K1 cells transfected with KLF13 plasmids or control for 48 h were harvested and resuspended in phosphate-buffered saline with a density of 1 × 10^7^ cell/ml. There were 0.1 mL of the above suspension subcutaneously injected at the buttock of 4-week-old male BALB/c athymic nude mice. The tumor volumes were recorded every week, and calculated with the formula below: V = length × (width)^2^/2. After inoculation of 5 weeks, while euthanizing the animals, tumor xenografts were weighted. The Institutional Animal Care and Use Committee of the Ethics Committee of National Cancer Center/National Clinical Research Center for Cancer/Cancer Hospital, Chinese Academy of Medical Sciences and Peking Union Medical College approved all test steps.

### Statistical analysis

Statistical analysis was made with GraphPad prism 8.0 software. All tests are shown as the mean ± standard deviation. Diversities between two groups were compared with student’s *t*-test, whereas diversities among multiple groups were compared with one way ANOVA followed by Tukey’s post hoc test. *P* < 0.05 suggested a statistically significant difference.

### Supplementary Information

**Additional file 1.** Supplementary figures.

**Additional file 2.** Supplementary table.

## Data Availability

The original contributions presented in the study are included in the article/Supplementary Material, further inquiries can be directed to the corresponding author/s.
