# Supplementary material for: Kruppel-like factor 13 acts as a tumor suppressor in thyroid carcinoma by downregulating IFIT1
Source: Biol Direct. 2023 Oct 10;18:65. doi: 10.1186/s13062-023-00422-5 (PMC10565980; doi:10.1186/s13062-023-00422-5)
Supplement: Supplementary file 1 — Additional file 1. Supplementary figures. [file 13062_2023_422_MOESM1_ESM.docx]

Figure S1


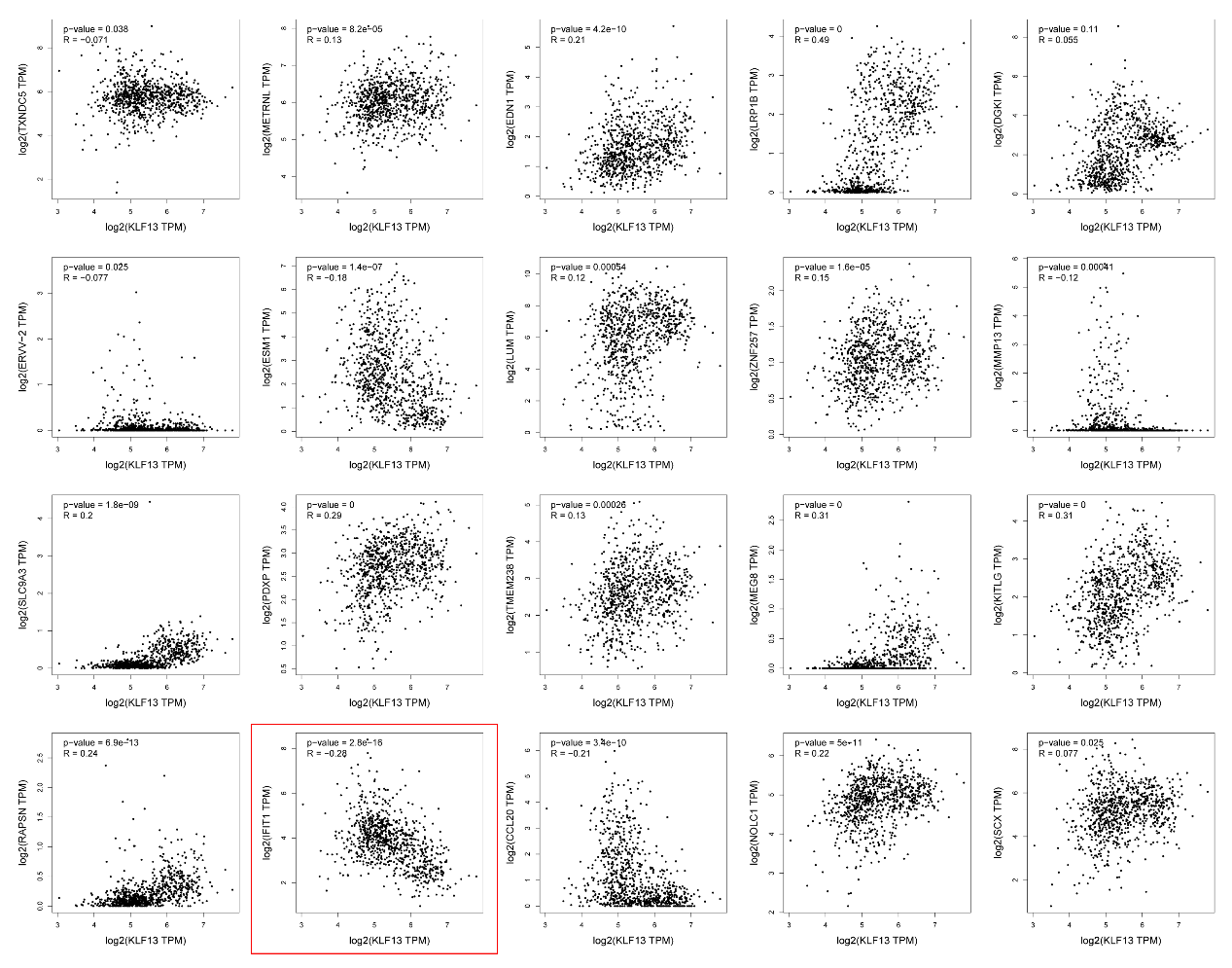


Figure S1: The correlation between KLF13 with targeted genes based on the TCGA database.

Figure S2


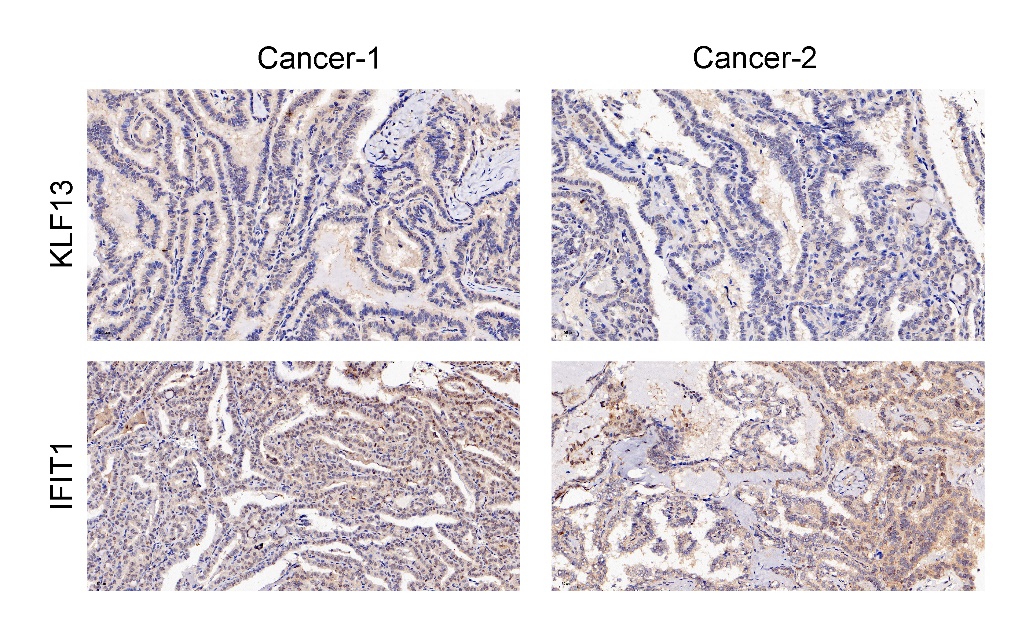


Figure S2: Typical IHC staining of KLF13 and IFIT1 in thyroid carcinoma.
