# Supplementary material for: Kruppel-like factor 13 acts as a tumor suppressor in thyroid carcinoma by downregulating IFIT1
Source: Biol Direct. 2023 Oct 10;18:65. doi: 10.1186/s13062-023-00422-5 (PMC10565980; doi:10.1186/s13062-023-00422-5)
Supplement: Supplementary file 2 — Additional file 2. Supplementary table. [file 13062_2023_422_MOESM2_ESM.docx]

Table S1 Spearman correlation analysis of expression between KLF13 and IFIT1 in 25 thyroid cancer tissues by IHC.

|  | KLF13 | |  |
| --- | --- | --- | --- |
|  | *r_s_* | *P* value |  |
| IFIT1 | -0.5732 | 0.0027 |  |

r, Spearman correlation
